# Occurrence and Diversity of Clinically Important *Vibrio* Species in the Aquatic Environment of Georgia

**DOI:** 10.3389/fpubh.2015.00232

**Published:** 2015-10-13

**Authors:** Tamar Kokashvili, Chris A. Whitehouse, Ana Tskhvediani, Christopher J. Grim, Tinatin Elbakidze, Nino Mitaishvili, Nino Janelidze, Ekaterine Jaiani, Bradd J. Haley, Nino Lashkhi, Anwar Huq, Rita R. Colwell, Marina Tediashvili

**Affiliations:** ^1^G. Eliava Institute of Bacteriophages, Microbiology, and Virology, Tbilisi, Georgia; ^2^United States Army Medical Research Institute of Infectious Diseases (USAMRIID), Fort Detrick, MD, USA; ^3^Institute for Advanced Computer Studies, University of Maryland College Park, College Park, MD, USA; ^4^Department of Cell Biology and Molecular Genetics, Maryland Pathogen Research Institute, University of Maryland College Park, College Park, MD, USA

**Keywords:** aquatic environment, Black Sea, lakes, infection, *Vibrios*, conventional culture, direct detection, diversity

## Abstract

Among the more than 70 different *Vibrio* species inhabiting marine, estuarine, and freshwater ecosystems, 12 are recognized as human pathogens. The warm subtropical climate of the Black Sea coastal area and inland regions of Georgia likely provides a favorable environment for various *Vibrio* species. From 2006 to 2009, the abundance, ecology, and diversity of clinically important *Vibrio* species were studied in different locations in Georgia and across seasons. Over a 33-month period, 1,595 presumptive *Vibrio* isolates were collected from the Black Sea (*n* = 657) and freshwater lakes around Tbilisi (*n* = 938). Screening of a subset of 440 concentrated and enriched water samples by PCR-electrospray ionization/mass spectrometry (PCR-ESI/MS) detected the presence of DNA from eight clinically important *Vibrio* species: *V. cholerae*, *V. parahaemolyticus, V. vulnificus, V. mimicus, V. alginolyticus, V. harveyi, V. metschnikovii*, and *V. cincinnatiensis*. Almost 90% of PCR/ESI-MS samples positive for *Vibrio species* were collected from June through November. Three important human-pathogenic *Vibrio* species (*V. cholerae, V. parahaemolyticus*, and *V. vulnificus*) were detected in 62.8, 37.8, and 21.4% of samples testing positive for *Vibrios*, respectively. The results of these activities suggest that natural reservoirs for human-pathogenic *Vibrios* exist in Georgian aquatic environments. Water temperature at all sampling sites was positively correlated with the abundance of clinically important *Vibrio* spp. (except *V. metschnikovii*), and salinity was correlated with species composition at particular Black Sea sites as well as inland reservoirs.

## Introduction

*Vibrio* species are ubiquitous and abundant in aquatic environments. They exist suspended in the water column, attached to plankton, and in the tissues or organs of various marine animals (1). The genus *Vibrio* includes more than 70 species, which are characterized as halophilic or non-halophilic according to their need for sodium chloride for growth (2, 3). At least 12 *Vibrio* species, including *V. cholerae*, *V. parahaemolyticus*, and *V. vulnificus*, are pathogenic for humans and important to public health (4–7).

Among more than 200 known *V. cholerae* serotypes, serotypes O1 and O139 have epidemic and clinical importance. These serotypes are the causative agents of cholera, a severe diarrheal disease with 50–60% mortality in untreated cases. Cholera is contracted through consumption of contaminated food and water. Non-O1 and non-O139 *Vibrio* serotypes (also called non-agglutinable or NAG, serotypes) have also been implicated as etiologic agents of moderate to severe gastroenteritis in humans (2, 8, 9). *V. parahaemolyticus*, a halophilic *Vibrio* species, mainly causes gastroenteritis associated with consumption of contaminated seafood. In the last decade, epidemics of gastroenteritis in Southeast Asia, Japan, and North America have been linked to *V. parahaemolyticus* pandemic serotype O3:K6 (10, 11). Wound infections and septicemia caused by *V. vulnificus* carry a case fatality rate (CFR) as high as 50% in healthy patients; the CFR of *V. vulnificus* infections is even higher among immuno*-*compromised patients or those with liver disease (6). *V. metschnikovii* can cause gastroenteritis and wound infections leading to septicemia, while *V. fluvialis*, *Grimontia hollisae* (formerly *V. hollisae*), and *V. furnissii* typically cause gastroenteritis (12, 13). *V. alginolyticus* is commonly associated with ear infections, but can also cause respiratory infections and bacteremia (14, 15). *Photobacterium damselae* subsp. *damselae* (formerly *V. damsela*) causes opportunistic wound infections that can rapidly progress to a necrotizing condition, and ultimately to sepsis, which is fatal in 30–50% of human cases (16). A few cases of human illness (e.g., septicemia, meningitis, and diarrhea) have been associated with *V. cincinnatiensis* (17). *V. alginolyticus*, *P. damselae*, and *V. harveyi* are also opportunistic pathogens of economic significance in aquaculture, responsible for high mortality in cultured fish and shellfish, sometimes destroying an entire aquaculture operation (18).

Prevention and control of infections caused by *Vibrio* species pathogenic for humans depend on understanding their ecology, pathogenicity, and modes of transmission. A warm, subtropical climate – such as in the Black Sea coastal region of the South Caucasus – may support growth and multiplication of most *Vibrio* species (2, 19–21). A limited number of short reports are available on the abundance of NAG *Vibrio* species (more specifically, *V. parahaemolyticus* and *V. alginolyticus*) in some coastal areas of the Black Sea (9, 22–24). In addition, environmental surveillance studies have been performed to fill gaps in the understanding of the ecology of *V. cholerae* and other *Vibrio* species of public health importance in Georgia and Azerbaijan (25–29). However, the full range of disease-causing *Vibrio* species in aquatic environments in this region has never been comprehensively examined.

The Black Sea coastal zones of Georgia and water reservoirs around Tbilisi have traditionally been popular recreational zones; the number of international visitors to the country has drastically increased in the last decade. The combination of climate change (in particular, elevated air and surface water temperatures) and the increasing anthropogenic effects of tourism may increase the risk of emergence and spread of water-borne and food-borne infections (30). An increase in the frequency of enteric diseases with diarrhea has been registered in Georgia, especially in the Ajara region, which is near the Black Sea sampling sites used in this study (31); a significant portion of these infections with idiopathic etiology may have been caused by pathogenic *Vibrio* species.

In light of these factors, the aim of this study was to conduct environmental surveillance to assess the abundance and diversity of clinically important *Vibrio* species along the Georgian coastal zone of the Black Sea and in freshwater reservoirs near Tbilisi. The study also aimed to evaluate the efficacy of conventional methods used by Georgian public health laboratories to detect and identify pathogenic *Vibrios* in the aquatic environment. To our knowledge, this report presents the first detailed description of environmental parameters in relation to the prevalence and community structure of pathogenic *Vibrio* species in recreational waters in Georgia.

## Materials and Methods

### Sample Sites and Collection

From June 2006 through October 2008, surface water samples were collected bi-weekly from July through September and monthly the rest of the year. Water samples were collected along the Georgian Black Sea coastal zone at four permanent stations located 50–100 m from the shore. Sample sites included two estuaries: Supsa (N 42°00.008′ E 41° 41.01′) and Chorokhi (N 41°36.116′ E 41° 34.021′); and two popular recreational/tourist attraction areas: Green Cape (N 41°41.91′ E 41° 42.01′) and Batumi Boulevard (N 41°39.570′ E 41°38.006′). Water samples were also collected 2–5 m from the shore of three inland reservoirs around Tbilisi: Kumisi Lake (Site 1: N 41°35.153′ E 044°51.591′; Site 2: N 41°34.839′ E 044°51.304′); Lisi Lake (Site 1: N 41°44.440′ E 044°44.261′; Site 2: N 41°44.483′ E 044°44.326′); and Tbilisi Sea (Site 1: N 41°46.150′ E 044°48.904′; Site 2: N 41°45.765′ E 044°50.308′). Sample locations are displayed in Figure S1 in Supplementary Material with coordinates listed in Table S1 in Supplementary Material.

Temperature, salinity, pH, conductivity, dissolved oxygen, and total dissolved solids were measured using a portable multi-log environmental meter (YSI Model 556 MPS, Yellow Springs, OH, USA), according to manufacturer’s instructions. The physicochemical parameters of each sample were measured in triplicate, and the mean was recorded for laboratory analysis; seasonal temperature and salinity measurements for the sampling sites are given in Table S2 in Supplementary Material. At the time of sample collection at each site, 100 L of water was filtered through plankton nets (200, 64, and 10-μm mesh size) to collect concentrated plankton samples and plankton-free water (32, 33).

### Bacterial Strains

Five *Vibrio* strains were obtained from the collection of the Institute Pasteur (CIP): *V. cholerae* CIP 62.13; *V. cholerae* CIP 55.91 (O1 classical); *V*. cholerae CIP *106855 (O1 El Tor)*; *V. cholerae* CIP 104151 (O139); and *V. natriegens* CIP 103193.

The National Center for Disease Control (NCDC) in Tbilisi, Georgia kindly provided two strains of *V. cholerae*: *V. cholerae* N145/P-1 (O1 classical) and *V. cholerae* N890/M-878 (O1 El Tor).

The following reference strains were obtained from the American Type Culture Collection (ATCC): *V. vulnificus* ATCC 27562; *V. parahaemolyticus* ATCC 17802; *V. metschnikovii* ATCC 700040; *V. fluvialis* ATCC 33809; *V. furnissii* ATCC 35016; *V. alginolyticus* ATCC 17749 serotype XII; *Photobacterium damselae* subsp. *damselae* ATCC 33539; *Grimontia* (*Vibrio*) *hollisae* ATCC 33564; *V. cincinnatiensis* ATCC 35912; and *V. harveyi* ATCC 14126.

### Isolation of *Vibrio* Species

Different volumes of plankton-free water (10–100 mL) were passed through 0.45-μm membrane filters to concentrate bacteria. The filters were placed onto thiosulfate-citrate-bile salts-sucrose (TCBS) agar, and incubated at 35°C for 24 h. After incubation, presumptive *Vibrio* colonies were counted. Filters were also incubated in 1% alkaline peptone water (APW), pH 8.6, for 18 h at 35°C to enhance growth of *Vibrios*. After incubation, a sterile inoculating loop was used to streak samples onto TCBS agar plates. Similarly, concentrated plankton samples were spread onto TCBS agar plates and/or inoculated in APW concentrate *Vibrios*. Plates were then incubated at 35–37°C for 24 h. Presumptive *Vibrio* colonies (yellow, green, and olive-green) that grew on TCBS plates were counted and sub-cultured onto T_1_N_1_ (1% trypticase, 1% sodium chloride) agar plates.

### Biochemical Identification of *Vibrio* Species Isolates

Presumptive *Vibrio* isolates were sub-cultured onto Luria-Bertani agar and screened for the following biochemical properties: gelatinase production; oxidase activity; salt requirement/tolerance (growth in T_1_N_1_–T_1_N_8_ solutions); glucose oxidation/fermentation; arginine dehydrolase utilization; lysine decarboxylase utilization; and utilization of sucrose, arabinose, lactose, and mannose (4, 32).

Biochemical identification parameters were analyzed using a software algorithm designed for this study. The algorithm was used to compare biochemical tests’ results for presumptive *Vibrio* isolates with those of 12 clinically important, well-characterized, standard strains of *Vibrio* species. The algorithm also included existing data on the physiological and biochemical properties of various *Vibrio* species (4, 33, 34). If the properties of a *Vibrio* isolate were similar to those of a reference strain, a percentage of affinity was calculated using different weighted factors for the particular biochemical parameter(s) (P, [P]).

### Identification of *Vibrio* Species Isolated by PCR

Bacterial DNA was extracted using an AquaPure genomic DNA isolation kit (Bio-Rad Laboratories, Hercules, CA, USA) according to manufacturer’s instructions. Species-specific PCR (internal transcribed spacer PCR) and collagenase-targeted PCR were used to confirm the identification of presumptive *V. cholerae, V. mimicus, V. parahaemolyticus, V. alginolyticus*, and *V. vulnificus* isolates (32, 33).

To determine the serogroup (O1 or O139) of *V. cholerae* isolates, a multiplex PCR was performed as described by Huq et al. (32, 33). The assay was also used to detect virulence factor genes (e.g., ctxA) in confirmed *V. cholerae* isolates. PCR primers and target genes used to detect clinically important *Vibrio* species are listed in Table S3 in Supplementary Material.

For identification of all other presumptive *Vibrio* species not identified by species-specific PCR, 16S rRNA was amplified and sequenced, and the sequences were compared to corresponding data in NCBI and RDPII databases (33).

### Direct Detection of *Vibrio* Species Isolates by PCR/ESI-MS

*Vibrio* species were directly detected in water samples with a *Vibrio*-specific PCR-electrospray ionization (ESI)/mass spectrometry (MS) assay (26). Briefly, total community DNA was amplified from water samples using an eight-reaction broad-range PCR assay targeted to members of the *Vibrio* genus and *Vibrionaceae* family. After PCR, a purified aliquot from each reaction was sprayed into a Bruker Daltonics microTOF (Billerica, MA, USA) mass spectrometer. Because of the high mass accuracy (mass measurement error <1 ppm) of the spectrometer, the mass of each PCR amplicon could be accurately determined and a base composition could be assigned with confidence (e.g., xA, xT, xC, and xG) (35). Because the assay included eight primers, multiple base counts were assigned to each sample from various parts of the genome. For confirmatory identification, base compositions of the samples were added to the Ibis database (National Center for Biotechnology Information, Bethesda, MD, USA) for comparison with base compositions of *Vibrio* reference strains and related bacteria.

### Direct Fluorescent Antibody Test

A direct fluorescent antibody (DFA) test was used to detect *V. cholerae* O1 and O139 in enriched and concentrated water and plankton samples (32, 33). Preparation of specimens was performed using a DFA test kit (New Horizon Diagnostics, Columbia, MD, USA) according to the manufacturer’s instructions. Specimens were examined with an epi-fluorescent microscope (Axioskop 40, Opton Zeiss, Germany) at 100× magnification.

### Data Analysis

Each water sample was tested in triplicate for all biochemical tests outlined above, and the mean values and standard errors were calculated for each variable on a given sampling date. For the purpose of analysis, seasons were defined as follows:

- *Winter*: December through February;
- *Spring*: March through May;
- *Summer*: June through August;
- *Fall*: September through November.

Statistical analysis was carried out using the Statistical Toolpak for Microsoft Excel 2010. Correlations (Pearson’s *r*) between factors described herein were significant at the 0.05 level.

## Results and Discussion

### Isolation and Phenotypic Characterization of Clinically Important *Vibrio* Species

During the study period, 1,440 water samples were collected and analyzed; from these, 1,595 presumptive *Vibrio* isolates were collected. Among these samples, 657 presumptive *Vibrio* isolates were collected from four sites on the Black Sea coast and 938 from three inland water reservoirs. Over 70% of the isolates were assigned to 10 clinically important *Vibrio* species based on biochemical characteristics. These isolates were recovered from concentrated and enriched water samples, as well as from plankton. Of the 1,595 isolates, 856 were identified as presumptive non-halophilic *Vibrios* (specifically, *V*. *cholerae* and *V*. *mimicus*) and 739 were identified as halophilic *Vibrio* species. As expected, the Black Sea *Vibrio* population was most the diverse: nine species of clinically important *Vibrios* were recovered (*V. parahaemolyticus*, *V. vulnificus*, *V. cholerae*, *V. metschnikovii*, *V. alginolyticus*, *V. harveyi*, *V. furnissii*, *V. fluvialis*, and *V. cincinnatiensis*). Only seven *Vibrio* species (*V. cholerae*, *V. vulnificus*, *V. mimicus*, *V. metschnikovii*, *V. alginolyticus*, *V. harveyi*, and *V. fluvialis*) were collected from the inland water reservoirs. In general, halophilic *Vibrio* species were more commonly found in the Black Sea coastal zones (47.8%), than in freshwater lakes (6.2%). Among the halophilic *Vibrio* species, *V. parahaemolyticus* was the most prevalent. *V. cholerae* comprised more than 65% of isolates from inland reservoirs, and 23% of those from Black Sea sites. It should be noted that up to 28% of isolates were identified only to genus level. In a separate study by Mitaishvili et al., some of these isolates were identified as non-human-pathogenic *Vibrio* species, including *V. natriegens*, *V. splendidus*, and *V. estuarinus* (36).

### PCR Identification of *Vibrio* Species

To establish the diversity of the *Vibrio* species more accurately and also for comparison purposes, a subset of 274 halophilic *Vibrio* isolates (attributed to eight *Vibrio* species, mostly *V. parahaemolyticus*) and 520 non-halophilic *Vibrio* isolates (512 *V. cholerae* and 8 *V. mimicus*) were subjected to either PCR with species-specific primers; or amplification, sequencing, and comparison of 16S rRNA signatures, employing data available in public databases. A high level of agreement (98.5%, or 503 isolates out of 512) was observed between standard biochemical and PCR identifications for *V. cholerae*. Similar results were observed among 144 isolates of *V. parahaemolyticus* (95.8%).

The agreement between the two methods was relatively low (38.5–46.5%) for other *Vibrio* species. For example, some of the *V. vulnificus* isolates presumptively identified based on biochemical analysis were found to be *V. cholerae* by PCR. The majority of presumptive *V. alginolyticus* and *V. cincinnatiensis* isolates were identified as *V. parahaemolyticus* by PCR. Additionally, several isolates of *V. fluvialis identified by biochemical tests* were determined to be *V. furnissii* by genetic analysis. These results most likely are due to the significant genetic variability of biochemical features (e.g., salt tolerance, sucrose, lactose, and arabinose utilization) and especially of the halophilic *Vibrio* species.

**Figure 1:**
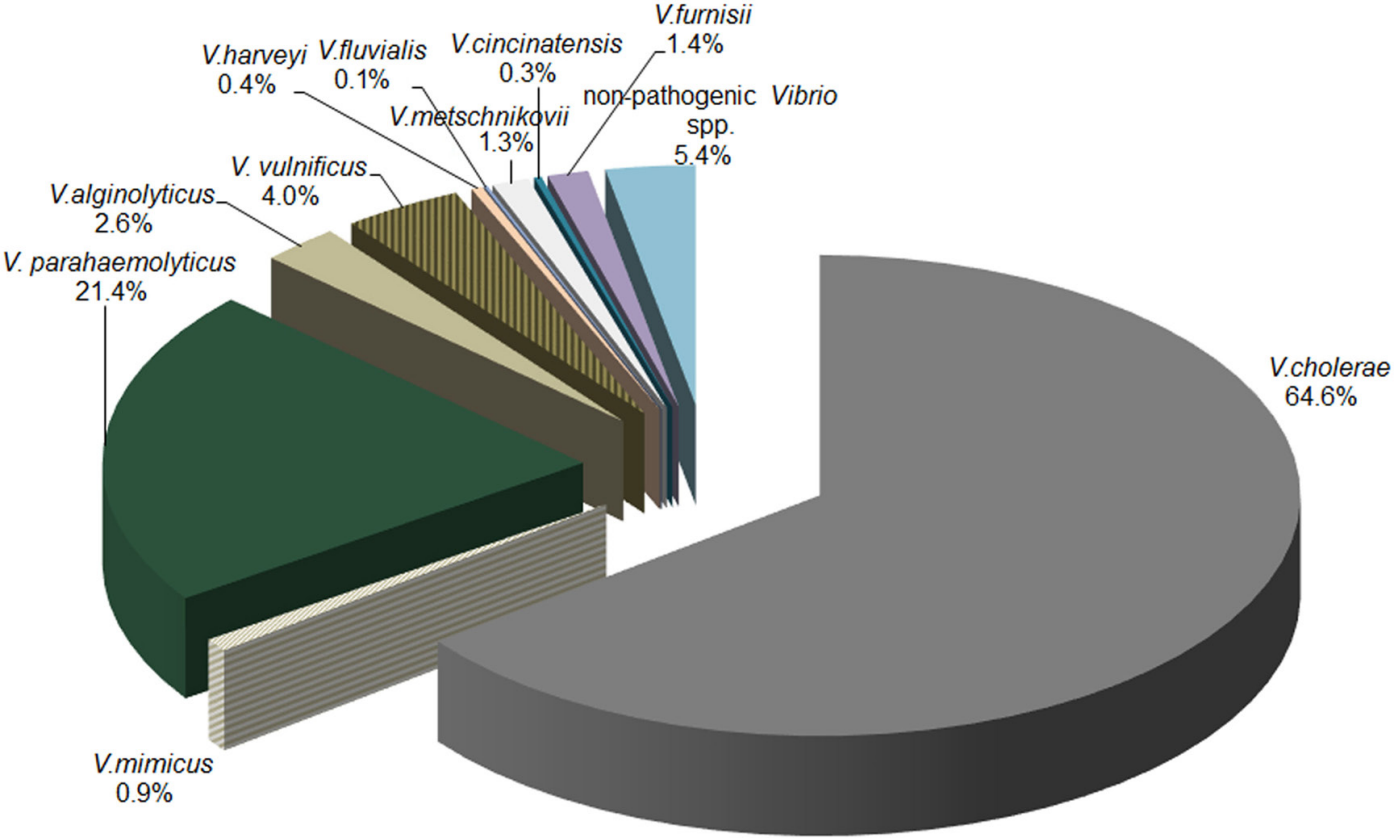
**Distribution of *Vibrio* species via PCR**. The distribution of *Vibrio* species detected by PCR in water samples collected across Georgia.

Among 794 presumptive *Vibrio* isolates, 10 of the clinically important *Vibrio* species were genetically identified. The distribution of species identified by biochemical analysis was different than that indicated by genetic testing. Among PCR-confirmed *Vibrio* isolates, *V. cholerae* was most prevalent (64.6%) among tested isolates, followed by *V. parahaemolyticus* (21.4%), *V. vulnificus* (4.0%), and *V. alginolyticus* (2.6%). Combined, the remaining six species (*V. metschnikovii*, *V. harveyi*, *V. furnissii*, *V. fluvialis*, *V. cincinnatiensis*, and *V. mimicus*) accounted for 4.4% of samples. Genetic identification was not definitive for 3% of the *Vibrio* isolates. The distributions of the 10 *Vibrio* species confirmed by PCR are presented in Figure 1.

### Direct Detection of Clinically Important *Vibrio* Species in Water Samples

In parallel with culture isolation, *Vibrio* species were directly detected in water samples collected during 2006–2008 from marine and freshwater sites (*n* = 440 samples; 220 from the Black Sea and 220 from lakes) using a PCR/ESI-MS method verified by Whitehouse et al. in a previous study employing standard and environmental *Vibrio* strains (26). Eight clinically important *Vibrio* species were detected by this method. Six of the eight *Vibrio* species were halophilic (*V. parahaemolyticus*, *V. vulnificus*, *V. alginolyticus*, *V. harveyi*, *V. metschnikovii*, and *V. cincinnatiensis*); the remaining two *Vibrio* species were non-halophilic (*V. cholerae* and *V. mimicus*), that is, not requiring additional salt for growth (all *Vibrios* have an absolute requirement for NaCl for growth). Two non-pathogenic *Vibrio* species [*V. natriegens* and *V. (Comamonas) neocistes*] were also detected.

**Figure 2:**
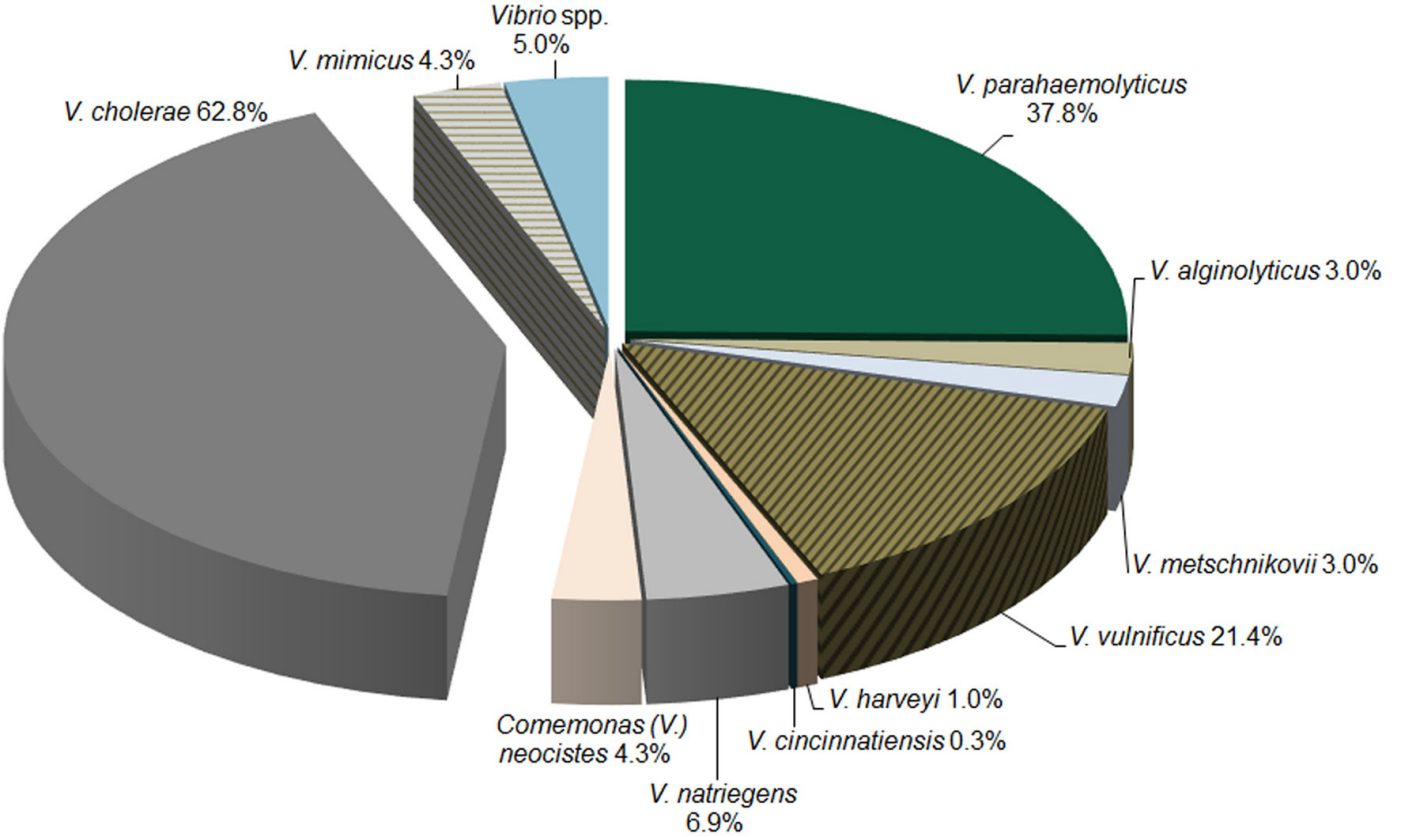
**Distribution of *Vibrio* species via PCR/ESI-MS**. The distribution of *Vibrio* species detected by PCR/ESI-MS in water samples collected across Georgia.

The distribution of samples positive for specific *Vibrio* species by PCR/ESI-MS is presented in Figure 2. In alignment with culture results, *V. cholerae* was the most prevalent pathogenic *Vibrio* species. In water samples containing the DNA of at least one *Vibrio* species, 62.8% contained *V. cholerae* (*n* = 304).

**Figure 3:**
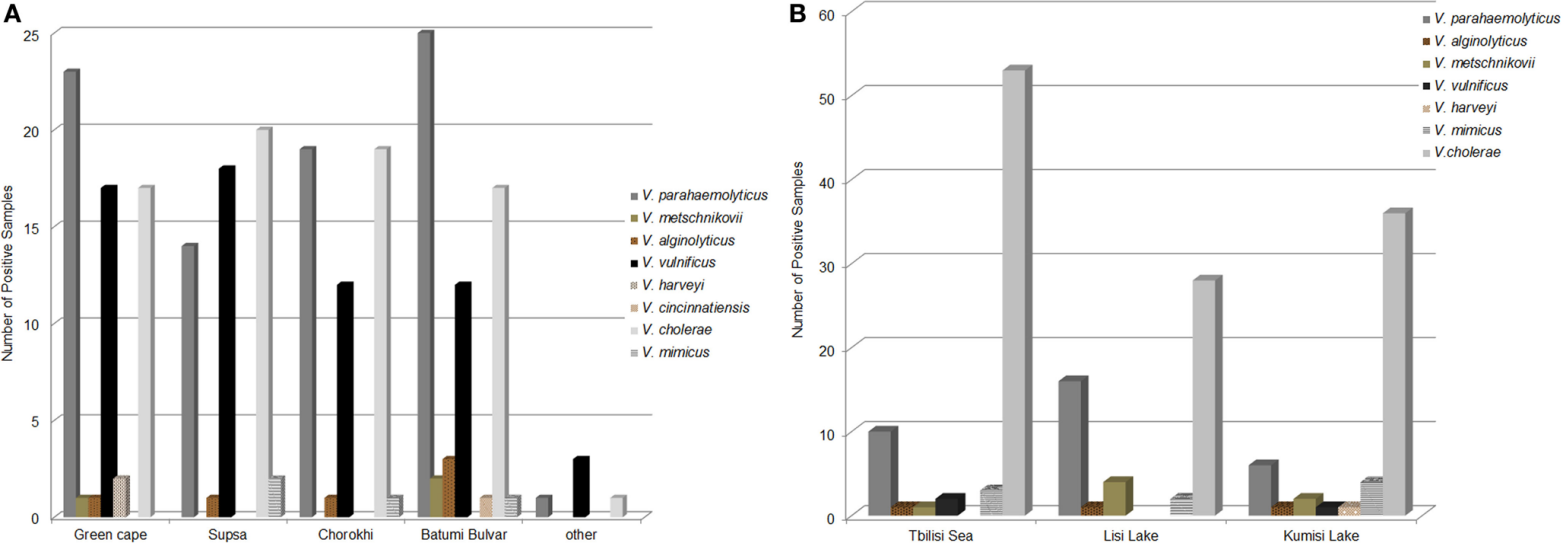
**Abundance of *Vibrio* species in Black Sea and lake water samples**. The abundance of *Vibrio* species in water samples as determined by PCR/ESI-MS from **(A)** The Black Sea and **(B)** Freshwater and brackish lakes.

Population diversity of Black Sea *Vibrio* species as determined by PCR/ESI-MS was similar to that observed among biochemically identified species (Figure 3A). Halophilic *Vibrio* species were more abundant in samples collected at Black Sea sites (72.6% of all *Vibrio-*positive samples) than those from freshwater lakes (29.1%); this is in agreement with culture results. Among *Vibrio*-positive Black Sea water samples (*n* = 153), *V. parahaemolyticus* was most frequently detected (53.6%), followed by *V. cholerae* and *V. vulnificus* (48.4 and 36.6%, respectively). Other *Vibrio* species (*V. alginolyticus, V. metschnikovii, V. fluvialis*, and *V. harveyi*) comprised between 0.7 and 3.9% of those identified in Black Sea samples.

Of the seven *Vibrio* species identified by conventional culture, six were detected in fresh and brackish lake samples, using PCR/ESI-MS (Figure 3B). *V. cholerae* was most frequently isolated from water samples collected at all three inland reservoirs (77.5% of all positive samples). DNA from other *Vibrio* spp. was detected less frequently, including: *V. parahaemolyticus* (21.2%), *V. mimicus* (6.0%), *V. metschnikovii* (4.6%), and *V. vulnificus* (2.0%). Among the lake samples, abundance and diversity of *Vibrio* species were highest in Kumisi Lake, with six species identified by phenotypic characterization and five by PCR. This diversity is most likely related to the relatively high salinity of the lake.

Also, in agreement with results of our previously published study, 20.7% of Black Sea and lake samples contained *V. natriegens* and/or *V. neocistes*. Also as observed in our previous report, *V. natriegens* was cultured from all four Black Sea sites along with other *Vibrio* species that are not pathogenic for humans (36).

### Seasonality in Abundance of Clinically Important *Vibrio* Species

**Figure 4:**
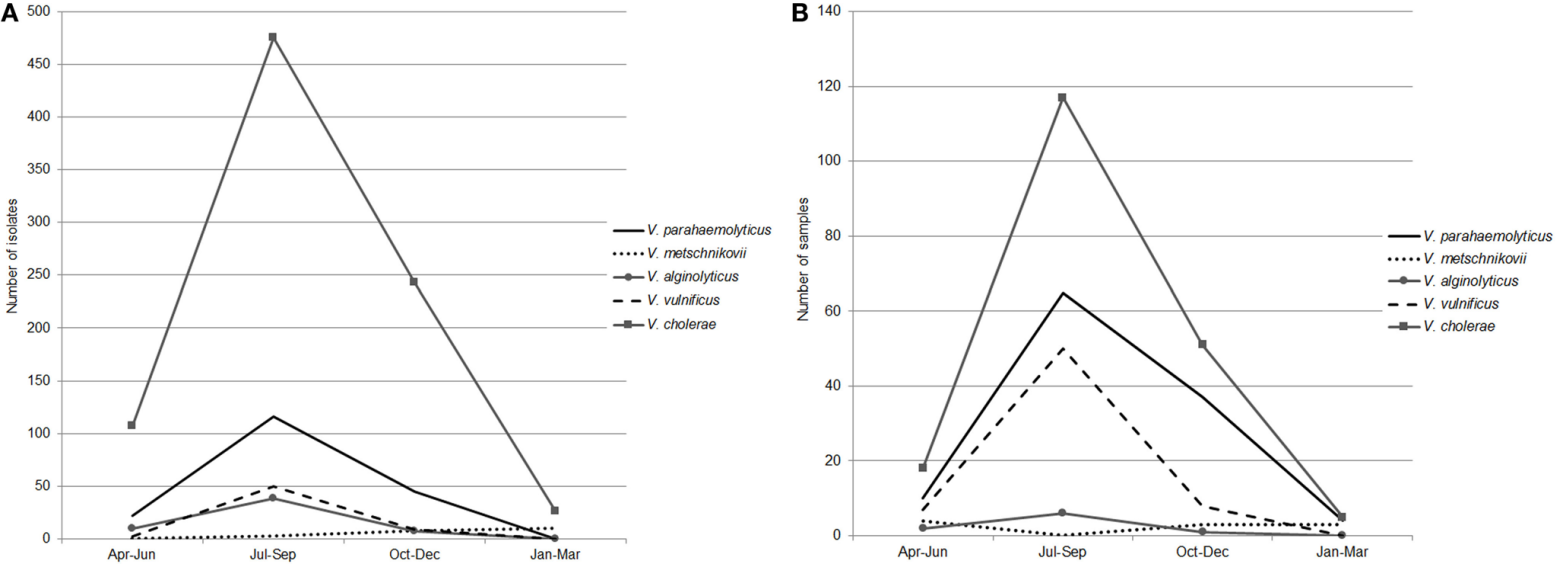
**Seasonal abundance of five *Vibrio* species**. The seasonal abundance of five *Vibrio* species (*V. parahaemolyticus*, *V. metschnikovii*, *V. alginolyticus*, *V. vulnificus*, and *V*. *cholerae*) as determined by **(A)** Biochemical identification and, **(B)** PCR/ESI-MS. Seasonal water temperature is reported in Table S2 in Supplementary Material.

Seasonal patterns in abundance of clinically important *Vibrio* species were reflected in the isolation frequency and results of PCR/ESI-MS. Ninety-five percent of *Vibrio* spp. were isolated from April through November, with the highest detection rates in July, August, and September (Figures 4A,B); water temperatures for these time periods (per location) are reported in Table S2 in Supplementary Material. In our earlier reports, we described a positive correlation between total *Vibrio* counts and water temperature at Black Sea and inland lake sites (37, 38). In addition, in the current study, we observed a correlation between isolation frequency of distinct groups of *Vibrio* species and water temperature at marine and freshwater sites. Interestingly, in this study, the correlation coefficient was higher for halophilic *Vibrio* species (*r* = 0.85 for Black Sea sites and *r* = 0.74 for lakes) than for *V. cholerae* (*r* = 0.45 for Black Sea sites and *r* = 0.64 for lakes). A different pattern of seasonal abundance was observed for *V. metschnikovii* isolates, which were collected during the cold season and were detected by PCR/ESI-MS from October through April (Figures 4A,B). This is indicative of cold tolerance of *V. metschnikovii* in comparison with the other pathogenic *Vibrio* species (39, 40).

### Salinity as an Environmental Factor Influencing *Vibrio* Community Composition

**Figure 5:**
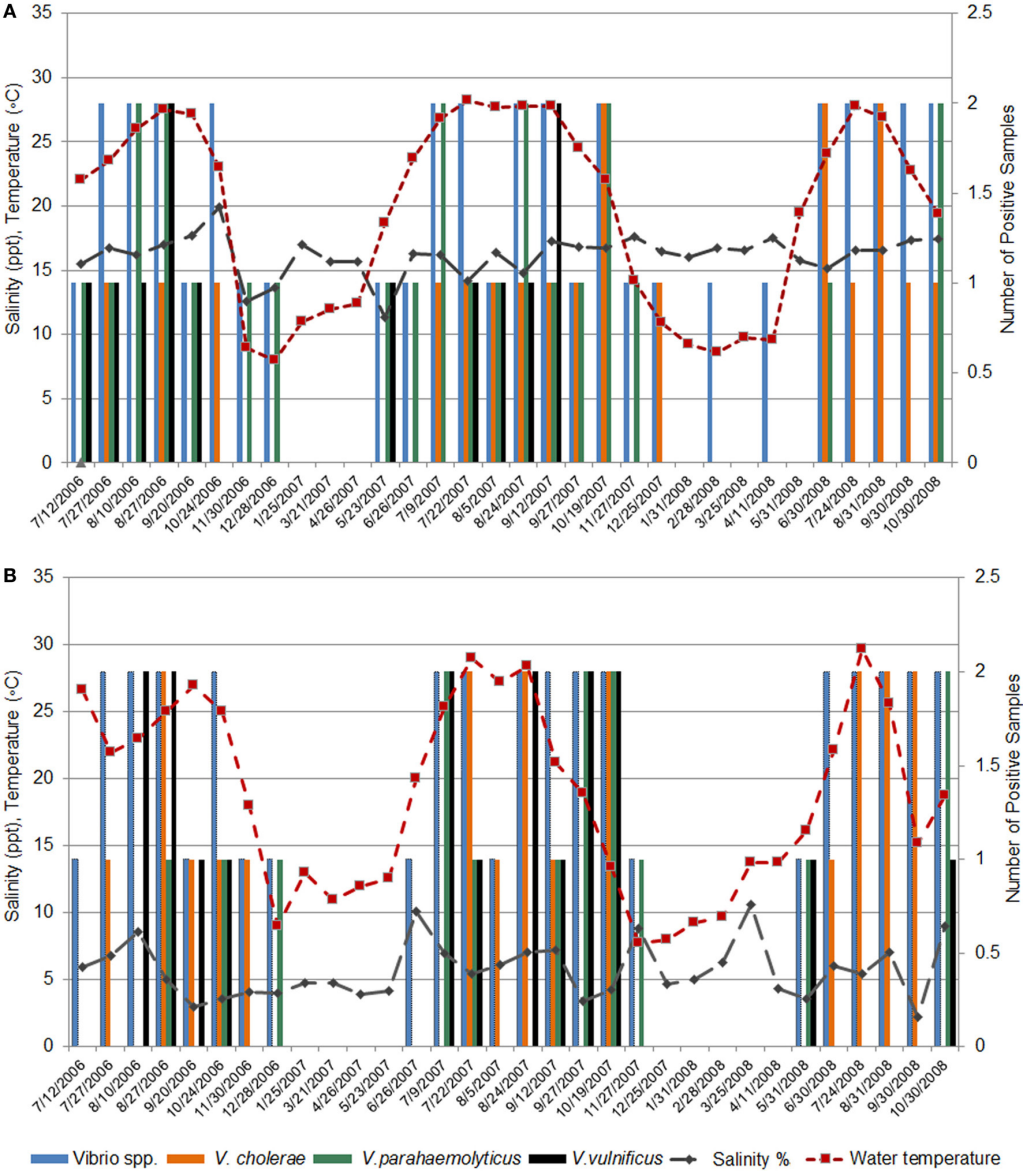
**Influence of water temperature on the detection of *Vibrio* species**. The influence of water temperature on the overall detection rates of *Vibrio* species by PCR/ESI-MS for **(A)** Batumi Boulevard, **(B)** Supsa estuary.

Salinity, an important environmental factor, can influence microbial community composition and abundance in aquatic environments (1, 2, 7). In an earlier published study, we reported no significant correlation between total *Vibrio* counts and salinity for Black Sea samples; instead, species diversity (by culturing and direct detection techniques) appears to be linked to salinity (37). For example, PCR/ESI-MS direct detection (Figure 3A) showed less diversity among *Vibrio* species in the Supsa and Chorokhi estuaries (five species at each site) than at the Green Cape and Batumi Boulevard sites (six and seven species, respectively). In the samples collected from Batumi Boulevard sites where the salinity is higher (16–20%), *V. parahaemolyticus* DNA was detected more frequently by PCR/ESI-MS than in samples from the Supsa estuary (salinity 3–10.6%), although a similar pattern of seasonal temperature dependence was observed at both Black Sea sampling stations (Figures 5A,B).

Among inland reservoirs, a more diverse *Vibrio* population was detected by both culture and PCR/ESI-MS in Kumisi Lake (five clinically important *Vibrio* species in total) than the other two lakes (Figure 3B). It was surprising to detect a number of pathogenic halophilic *Vibrio* species in inland lakes, where non-halophilic *Vibrios* were predicted (and ultimately isolated) to be more prevalent; for example, *V. alginolyticus* was detected in these reservoirs, and was most abundant in the Tbilisi Sea (average salinity of 0.1%). This may be explained by the proximity of this artificially created reservoir (fed by the Iori River) to three small saltwater lakes that existed in the area before the creation of the Tbilisi Sea in the 1950s (41).

### *V. cholerae*: A Prevalent Human-Pathogenic *Vibrio* in Georgian Aquatic Environments

The most important human-pathogenic *Vibrio* species, *V. cholerae*, was demonstrated by culture and direct detection to be prevalent in Georgian aquatic environments. According to PCR/ESI-MS, *V. cholerae* was present in the majority of lake samples (77.5% of positive samples). *V. cholerae* was detected less frequently in Black Sea samples (48.4%). This aligned with culture results, among which *V. cholerae* comprised 23 and 75.2% of *Vibrio* species isolates from the Black Sea and lake samples, respectively. According to our earlier report, the vast majority (94.6%) of Georgian isolates of *V. cholerae* were attributed to the non-O1/non-O139 group (28). These groups are generally non-pathogenic to humans, although some serotypes can cause mild to severe gastroenteritis (8, 9).

**Figure 6:**
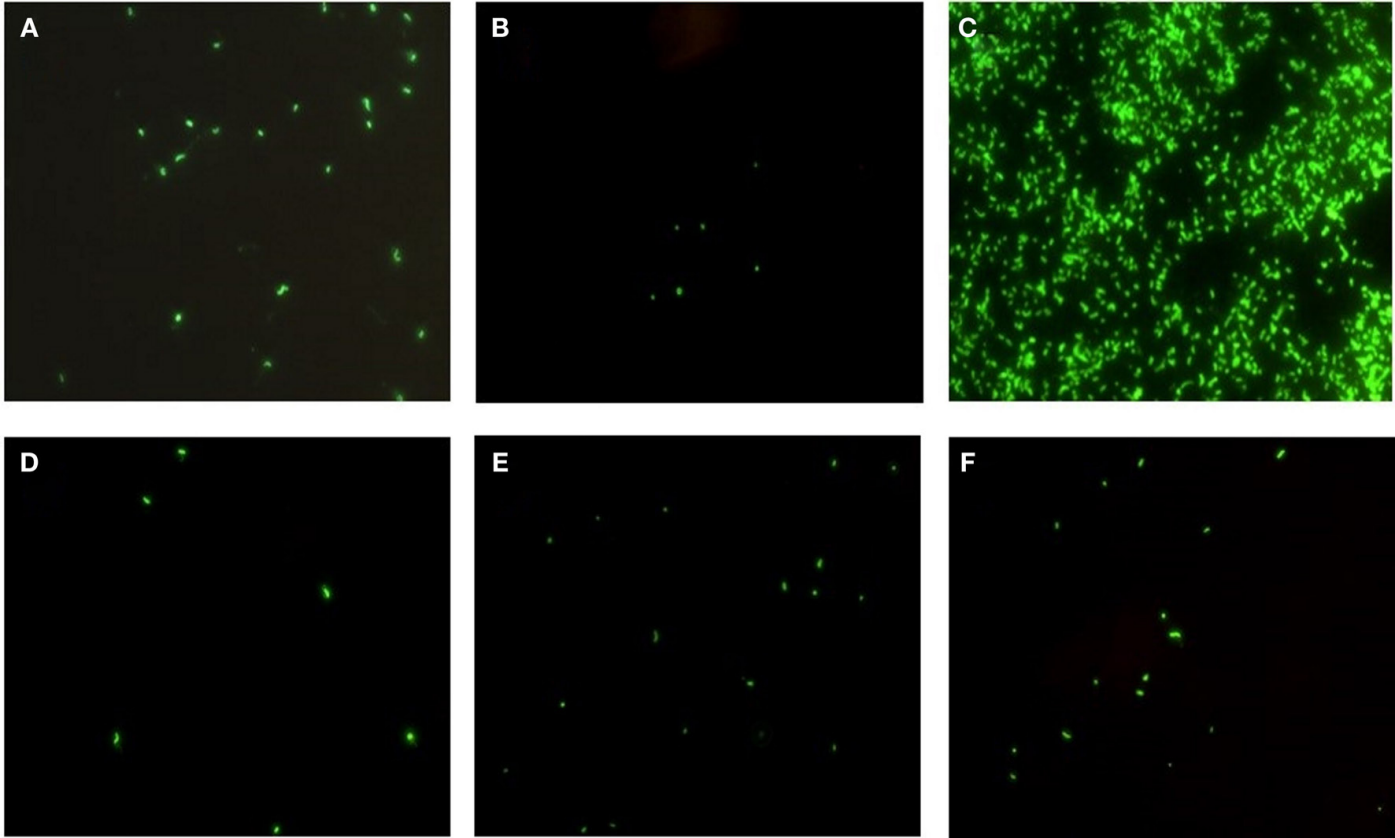
**Detection of *V. cholerae* O1 by DFA in Black Sea and lake samples**. Detection of *V. cholerae* O1 in water samples from **(A–C)** Green Cape sample sites on the Black Sea, and **(D–F)** Kumisi Lake. Concentrated water samples are shown in **(A,D)**, plankton samples in **(B,E)** and enriched water samples in **(C,F)**. Organisms were detected and visualized using a direct fluorescent-monoclonal antibody (DFA) kit.

**Figure 7:**
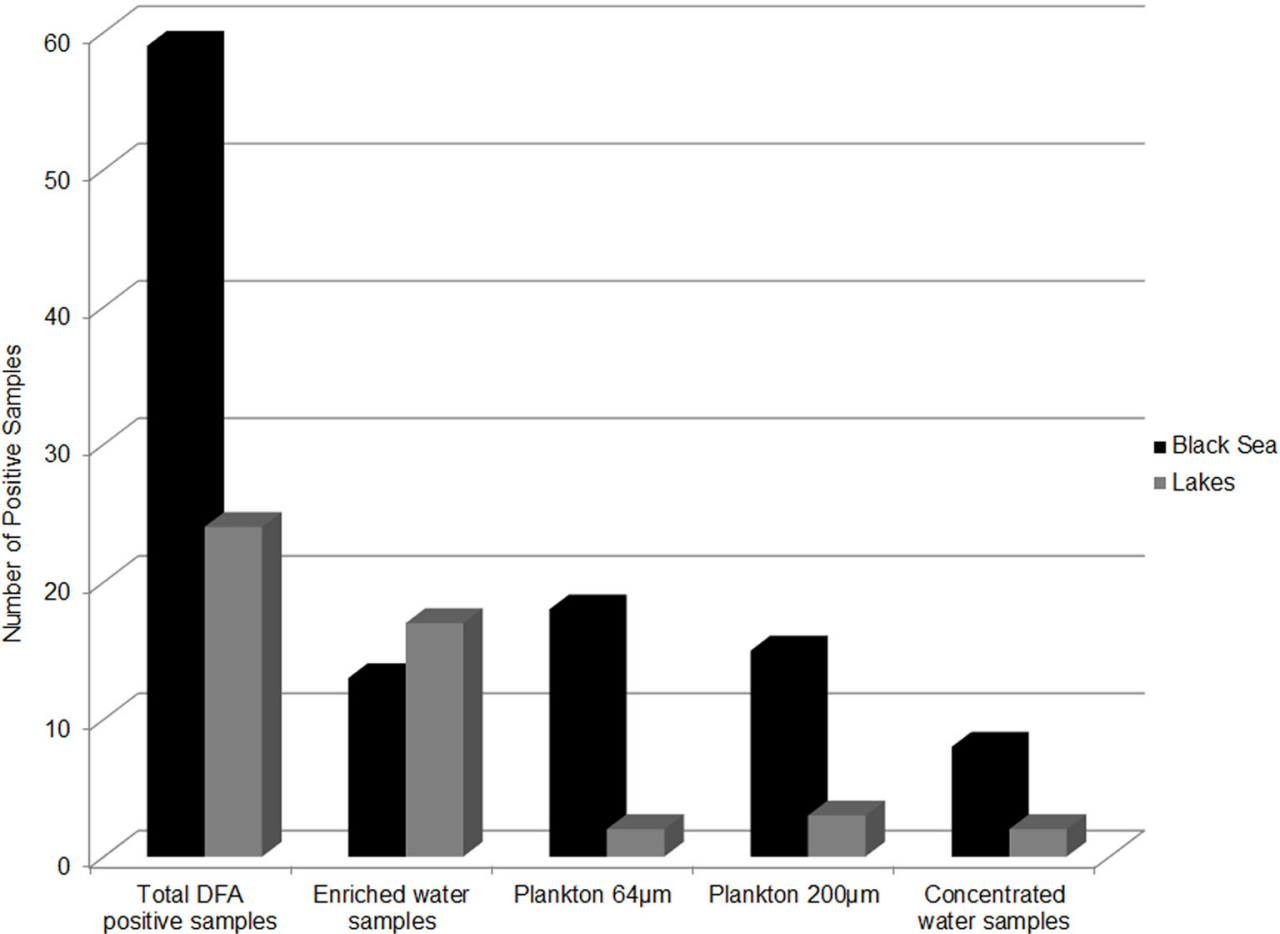
**Distribution of *V. cholerae* O1 DFA positive samples**. The distribution by sample type and sampling site of *V. cholerae* O1 positive samples as detected by DFA.

Epidemic *V. cholerae* serotype O1 was directly detected by DFA in the concentrated and enriched water and plankton samples collected at all four sites in the coastal zone of the Black Sea during the study period (Figure 6). Toxigenic *V. cholerae* O1 was also detected in lake samples (specifically from Kumisi Lake and the Tbilisi Sea) collected in 2008 and 2009 (Figure 6). In addition, signals indicative of *V. cholerae* O139 were detected in eight marine samples collected in the summer of 2008. These data, in combination with results of our earlier DFA investigations on lake samples collected in 2006 and 2007, indicate that toxigenic *V. cholerae* is common in all of the target water bodies in this study (25). These serotypes were detected most frequently in Black Sea samples and often associated with plankton fractions (Figure 7). This is in agreement with previously published serological data and multiplex PCR data from other investigators studying *V. cholerae* (28). Forty-six isolates (most of which were collected from enriched marine samples) were confirmed as *V. cholerae* serotype O1 and six isolates were presumptively identified as *V. cholerae* serotype O139. Some of the *V. cholerae* O1 strains belonged to the El Tor biotype, while others revealed characteristics of hybrid variants. Two O1 and six non-O1 strains carried ctx genes.

Interestingly, *V. cholerae* was observed along with two other important *Vibrio* species in the same ecological niche: *V. parahaemolyticus* (33.1% of PCR-ESI/MS samples) and *V. vulnificus* (11.6%). The increased probability of co-mingling between *V. cholerae* and these important *Vibrio* species could facilitate transfer of genetic material (e.g., virulence factors) between the species.

### Comparative Ecology of *V. parahaemolyticus* in Marine and Lake Reservoirs

**Figure 8:**
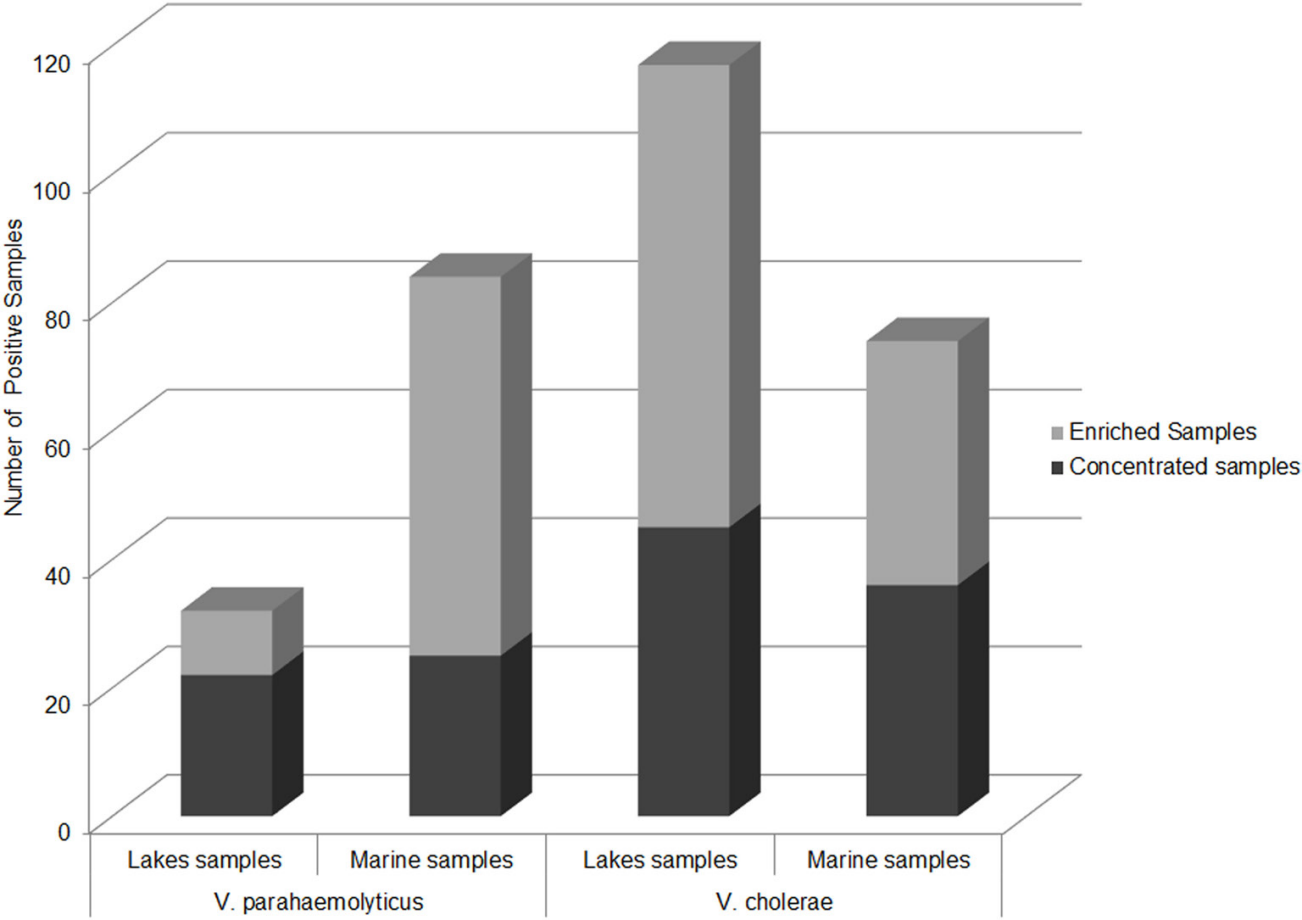
**Abundance of PCR/ESI-MS positive samples of *V. parahaemolyticus* and *V. cholerae***. Number of samples by sample type and sample site that yielded detectable levels of *V. parahaemolyticus* and *V. cholerae* via PCR/ESI-MS.

The second most important human-pathogenic *Vibrio* species, *V. parahaemolyticus*, was detected by PCR/ESI-MS in samples from the Black Sea. Most surprisingly, *V. parahaemolyticus* was detected in all sampled lakes, but no confirmed *V. parahaemolyticus* isolates were recovered from these sites. Interestingly, *V. parahaemolyticus* was more frequently detected by PCR/ESI-MS in concentrated lake samples than enriched lake samples; the reverse of this was observed for Black Sea samples (Figure 8). For comparison, as expected, DNA from *V. cholerae* was found most frequently in enriched lake samples, and in equal quantities in enriched and concentrated marine samples. The abundance of *V. parahaemolyticus* DNA in fresh and brackish water sites is likely explained by the lower salinity of lake water, which is a stressor for *V. parahaemolyticus*, a moderately halophilic bacterium. The low frequency of isolation of *V. parahaemolyticus* from lake water, coupled with the relatively high frequency of direct molecular detection, suggests that the species may be present in a viable but non-culturable state. Isolation of *V. parahaemolyticus*-specific bacteriophages from Lake Kumisi and Lake Lisi samples also supports the conclusion that this *Vibrio* species is present (42). The possibility of *V. parahaemolyticus* existing in a transitional state in freshwater reservoirs, in association with freshwater fish, has been proposed by Sarkar et al. and other researchers and is important, as this may serve as a potential exposure route to the bacterium (43, 44).

## Conclusion

In this study, we describe the occurrence, diversity, and seasonal distribution of clinically important *Vibrio* species in recreational waters in the South Caucasus. In total, 10 clinically important *Vibrio* species (of 12 known species) were detected among these sites; nine of which were detected in Black Sea coastal waters and six in inland sites. The Black Sea *Vibrio* populations were dominated by *V. parahaemolyticus*, followed by *V. cholerae*, *V. vulnificus*, and *V. alginolyticus*. *V. cholerae* was readily detected in fresh and brackish water samples collected at sites near Tbilisi.

*V. cholerae* was observed in the same ecological niche as two other important *Vibrio* species: *V. vulnificus* and *V. alginolyticus*. This finding is significant for several reasons. First, the possibility of acquiring infections caused by these pathogens will likely increase as the concentration/diversity of pathogenic *Vibrio* species located in a single niche increases. Second, such cohabitation increases the opportunity for interspecies interactions and potential exchanges of genetic material such as virulence factor genes.

As observed in previous studies, isolation of target *Vibrio* species and their direct detection using DNA-based methods for detection in marine and lake samples revealed seasonal patterns related to temperature that suggested that temperature affects *Vibrio* abundance, while salinity affects *Vibrio* species composition (37, 38).

Data obtained by conventional bacteriology, DFA, and PCR/ESI-MS analysis indicate that environmental reservoirs for toxigenic *V. cholerae* O1 (possibly O139 at some marine sites) are present in the recreational waters of Georgia, including freshwater lakes near Tbilisi, and the Georgian coastal zone of the Black Sea. Of particular note is the detection of *V. cholerae* O1 in the Tbilisi Sea, a freshwater reservoir that serves as a source of drinking water for several districts of Tbilisi. Previous detection of pandemic variants of *V. parahaemolyticus* (serotypes O3:K6) suggested increased risk for infection with the organism in areas where it is detected but not known to have (or that are not monitored for) outbreaks of seafood-related gastroenteritis or other water-related illnesses (28). This is even more apparent when the high frequency of seasonally reported enteric diseases with idiopathic etiologies in Georgia (especially in the Ajara region) is taken into account (30, 31).

Perpetually changing environmental conditions (e.g., increasing surface water temperatures) can significantly elevate the risk of infections related to potentially human-pathogenic *Vibrio* species. Such changes may also affect temperate regions with mild subtropical climates such as the South Caucasus. In the South Caucasus, climate change may lead to accelerated ecological problems and raise additional public health issues. In the coming decade, increases in air temperature (with associated long-lasting hot summers) and frequent heavy storms, combined with elevated surface water temperatures (up to ≥30°C) predicted for the Black Sea coastal areas, could trigger massive mortalities of aquatic organisms and possibly a significant rise in the incidence of diarrheal disease (30). Similarly, we can expect to observe such effects of climate change (with corresponding consequences for public health) associated with the recreational water bodies around Tbilisi.

In summary, although the occurrence of potentially pathogenic autochthonous water bacteria such as *Vibrios* cannot be controlled in natural ecosystems, the likelihood of the incidence of human illness can be reduced. This can be accomplished by limiting exposure to recreational water bodies with suspected elevated numbers of pathogenic *Vibrio* species. Furthermore, exposure can also be limited in areas where changes in ecological parameters have the potential to trigger proliferation of clinically important *Vibrio* species. Therefore, regular monitoring of water reservoirs for possible microbial pathogens is recommended to allow for early response by public health authorities (e.g., prevention and treatment measures to combat relevant diseases). The results presented in this report, as well as those in our previous publications (17, 27, 30) provide a foundation for effective monitoring of target *Vibrio* species, as well as for creation of predictive models based on environmental indicators.

## Conflict of Interest Statement

The authors declare that the research was conducted in the absence of any commercial or financial relationships that could be construed as a potential conflict of interest.

## Supplementary Material

The Supplementary Material for this article can be found online at http://journal.frontiersin.org/article/10.3389/fpubh.2015.00232
