# Supplementary material for: Occurrence and Diversity of Clinically Important Vibrio Species in the Aquatic Environment of Georgia
Source: Front Public Health. 2015 Oct 13;3:232. doi: 10.3389/fpubh.2015.00232 (PMC4603242; doi:10.3389/fpubh.2015.00232)
Supplement: Supplementary file 1 [file Table_1.DOCX]

***Supplementary Material***

**Abundance and diversity of clinically important *Vibrio species* in the aquatic environments of Georgia**

**Tamar Kokashvili^1^, Chris A. Whitehouse^2**^, Ana Tskhvediani^1^, Christopher J. Grim^3,4***^, Tinatin Elbakidze^1^_,_ Nino Mitaishvili^1****^, Nino Janelidze^1^, Ekaterine Jaiani^1^, Bradd Haley^4^, Nino Lashkhi^1^, Anwar Huq^4^, Rita R. Colwell^3,4^, Marina Tediashvili^1*^**

^1^G. Eliava Institute of Bacteriophages, Microbiology and Virology, Tbilisi, Georgia

^2^United States Army Medical Research Institute of Infectious Diseases (USAMRIID), Fort Detrick, MD, USA ^3^Institute for Advanced Computer Studies, University of Maryland, College Park, MD, USA ^4^Maryland Pathogen Research Institute, Department of Cell Biology and Molecular Genetics, University of Maryland, College Park, MD, USA

*** Correspondence:** M. Tediashvili, G. Eliava Institute of Bacteriophages, Microbiology and Virology, 3 Gotua Street, Tbilisi, 0160, Georgia.

[m_tediash.ibmv@caucasus.net](mailto:m_tediash.ibmv@caucasus.net)

* Opinions, interpretations, conclusions and recommendations are those of the author and are not necessarily endorsed by the US Army.

*** Current address: U.S. Food and Drug Administration, Laurel, MD U.S.A.

**** Current address: Branch of Battelle Memorial Institute in Georgia; 0105 Tbilisi, Georgia

**Supplementary Data**

1. **Supplementary Figures and Tables**

## Supplementary Tables

**Supplementary Table 1. Aquatic sample sites used in this study.** Sample sites listed by sample location type (e.g., estuary, recreational, inland), sample location, and sample coordinates.

| **Sample Location Type** | **Sample Site** | | **Coordinates** |
| --- | --- | --- | --- |
| Estuaries | Supsa | | N 42º00.008’ E 41º 41.01’ |
|  | Chorokhi | | N 41º36.116’ E 41º 34.021’ |
| Recreational Areas | Green Cape | | N 41º41.91’ E 41º 42.01’ |
|  | Batumi Boulevard | | N 41º39.570’ E 41º38.006' |
| Inland Reservoirs | Kumisi Lake | Site 1 | N 41°35.153' E 044°51.591' |
|  |  | Site 2 | N 41°34.839' E 044°51.304' |
|  | Lisi Lake | Site 1 | N 41°44.440' E 044°44.261' |
|  |  | Site 2 | N 41°44.483' E 044°44.326' |
|  | Tbilisi Sea | Site 1 | N 41°46.150' E 044°48.904' |
|  |  | Site 2 | N 41°45.765' E 044°50.308' |
