# Supplementary material for: Occurrence and Diversity of Clinically Important Vibrio Species in the Aquatic Environment of Georgia
Source: Front Public Health. 2015 Oct 13;3:232. doi: 10.3389/fpubh.2015.00232 (PMC4603242; doi:10.3389/fpubh.2015.00232)
Supplement: Supplementary file 2 [file Table_2.DOCX]

***Supplementary Material***

**Abundance and diversity of clinically important *Vibrio species* in the aquatic environments of Georgia**

**Tamar Kokashvili^1^, Chris A. Whitehouse^2**^, Ana Tskhvediani^1^, Christopher J. Grim^3,4***^, Tinatin Elbakidze^1^_,_ Nino Mitaishvili^1****^, Nino Janelidze^1^, Ekaterine Jaiani^1^, Bradd Haley^4^, Nino Lashkhi^1^, Anwar Huq^4^, Rita R. Colwell^3,4^, Marina Tediashvili^1*^**

^1^G. Eliava Institute of Bacteriophages, Microbiology and Virology, Tbilisi, Georgia

^2^United States Army Medical Research Institute of Infectious Diseases (USAMRIID), Fort Detrick, MD, USA ^3^Institute for Advanced Computer Studies, University of Maryland, College Park, MD, USA ^4^Maryland Pathogen Research Institute, Department of Cell Biology and Molecular Genetics, University of Maryland, College Park, MD, USA

*** Correspondence:** M. Tediashvili, G. Eliava Institute of Bacteriophages, Microbiology and Virology, 3 Gotua Street, Tbilisi, 0160, Georgia.

[m_tediash.ibmv@caucasus.net](mailto:m_tediash.ibmv@caucasus.net)

* Opinions, interpretations, conclusions and recommendations are those of the author and are not necessarily endorsed by the US Army.

*** Current address: U.S. Food and Drug Administration, Laurel, MD U.S.A.

**** Current address: Branch of Battelle Memorial Institute in Georgia; 0105 Tbilisi, Georgia

**Supplementary Data**

1. **Supplementary Figures and Tables**

## Supplementary Tables

**Supplementary Table 2. Seasonal variability of water temperature and salinity.** Temperature and salinity values across seasons at each sampling site.

|  | | | **Black Sea** | | | | **Inland Reservoirs** | | |
| --- | --- | --- | --- | --- | --- | --- | --- | --- | --- |
|  |  |  | **Supsa**  **Estuary** | **Batumi Boulevard** | **Green Cape** | **Chorokhi**  **Estuary** | **Lisi Lake** | **Tbilisi Sea** | **Kumisi Lake** |
| **Water temperature (^o^C)** | Mar -May | min | 9.7 | 9.6 | 10.4 | 10.0 | 9.5 | 5.1 | 8.0 |
|  |  | max | 16.1 | 19.5 | 20.6 | 19.1 | 18.1 | 19.0 | 21.4 |
|  |  | average | 12.5 | 13.7 | 14.7 | 14.2 | 13.2 | 11.8 | 14.7 |
|  | Jun -Aug | min | 22 | 22.0 | 22.0 | 22.0 | 22.7 | 17.6 | 23.0 |
|  |  | max | 29.7 | 28.2 | 28.5 | 28.8 | 29.0 | 27.0 | 28.7 |
|  |  | average | 25.5 | 26.0 | 26.0 | 25.1 | 25.8 | 23.9 | 26.1 |
|  | Sep - Nov | min | 9.0 | 9.0 | 10.0 | 8.0 | 8.6 | 12.0 | 8.0 |
|  |  | max | 27.6 | 27.8 | 28.0 | 26.7 | 26.0 | 24.0 | 25.0 |
|  |  | average | 19.3 | 21.7 | 21.4 | 20.6 | 18.7 | 19.4 | 18.6 |
|  | Dec - Feb | min | 7.7 | 8.0 | 8.6 | 8.3 | 1.1 | 4.4 | 2.0 |
|  |  | max | 13.0 | 10.9 | 13.0 | 11.0 | 5.0 | 9.0 | 7.0 |
|  |  | average | 9.5 | 9.5 | 10.3 | 9.2 | 3.4 | 7.2 | 4.5 |
| **Salinity ‰** | Mar -May | min | 3.6 | 11.3 | 11.1 | 8.0 | 1.4 | 0.1 | 3.0 |
|  |  | max | 10.6 | 17.6 | 16.6 | 14.5 | 1.7 | 0.2 | 3.7 |
|  |  | average | 5.2 | 15.4 | 14.9 | 12.1 | 1.6 | 0.2 | 3.4 |
|  | Jun -Aug | min | 3.0 | 14.2 | 11.2 | 3.2 | 1.6 | 0.1 | 3.0 |
|  |  | max | 10.1 | 17.0 | 17.1 | 17.0 | 1.9 | 0.2 | 4.7 |
|  |  | average | 6.4 | 15.9 | 15.1 | 9.4 | 1.7 | 0.1 | 3.6 |
|  | Sep - Nov | min | 2.2 | 12.2 | 16.1 | 6.1 | 1.6 | 0.1 | 3.4 |
|  |  | max | 8.8 | 19.9 | 20.8 | 16.8 | 2.0 | 0.2 | 4.7 |
|  |  | average | 5.8 | 17.0 | 17.6 | 12.2 | 1.8 | 0.1 | 3.9 |
|  | Dec - Feb | min | 4.7 | 13.6 | 15.7 | 7.0 | 1.2 | 0.1 | 2.9 |
|  |  | max | 6.3 | 16.7 | 17.3 | 12. | 1.7 | 0.2 | 3.7 |
|  |  | average | 5.2 | 16.0 | 16.5 | 10.9 | 1.6 | 0.1 | 3.4 |
