# Supplementary material for: Occurrence and Diversity of Clinically Important Vibrio Species in the Aquatic Environment of Georgia
Source: Front Public Health. 2015 Oct 13;3:232. doi: 10.3389/fpubh.2015.00232 (PMC4603242; doi:10.3389/fpubh.2015.00232)
Supplement: Supplementary file 3 [file Table_3.DOCX]

***Supplementary Material***

**Abundance and diversity of clinically important *Vibrio species* in the aquatic environments of Georgia**

**Tamar Kokashvili^1^, Chris A. Whitehouse^2**^, Ana Tskhvediani^1^, Christopher J. Grim^3,4***^, Tinatin Elbakidze^1^_,_ Nino Mitaishvili^1****^, Nino Janelidze^1^, Ekaterine Jaiani^1^, Bradd Haley^4^, Nino Lashkhi^1^, Anwar Huq^4^, Rita R. Colwell^3,4^, Marina Tediashvili^1*^**

^1^G. Eliava Institute of Bacteriophages, Microbiology and Virology, Tbilisi, Georgia

^2^United States Army Medical Research Institute of Infectious Diseases (USAMRIID), Fort Detrick, MD, USA ^3^Institute for Advanced Computer Studies, University of Maryland, College Park, MD, USA ^4^Maryland Pathogen Research Institute, Department of Cell Biology and Molecular Genetics, University of Maryland, College Park, MD, USA

*** Correspondence:** M. Tediashvili, G. Eliava Institute of Bacteriophages, Microbiology and Virology, 3 Gotua Street, Tbilisi, 0160, Georgia.

[m_tediash.ibmv@caucasus.net](mailto:m_tediash.ibmv@caucasus.net)

* Opinions, interpretations, conclusions and recommendations are those of the author and are not necessarily endorsed by the US Army.

*** Current address: U.S. Food and Drug Administration, Laurel, MD U.S.A.

**** Current address: Branch of Battelle Memorial Institute in Georgia; 0105 Tbilisi, Georgia

**Supplementary Data**

1. **Supplementary Figures and Tables**

## Supplementary Tables

**Supplementary Table 3. PCR Primers and targets.** PCR primers and target genes specific for detection selected clinically important *Vibrio* species.

| **Species** | **Target** | **Primer** | **Sequence (5’-3’)** | **Reference** |
| --- | --- | --- | --- | --- |
| *V. parahaemolyticus* | Collagenase -targeted | VA-F | CGAGTACAGTCACTTGAAAGC | Di Pinto *et al.* 2006 |
|  |  | VA-R | CACAACAGAACTCGCGTTACC |  |
| *V. cholerae* | ITS | PVC-F2 | TTAAGCSTTTTCRCTGAGAATG | Huq *et al.* 1999 |
|  |  | PVCM-R1 | AGTCACTTAACCATACAACCCG |  |
|  | *ctxA* | PCTA-94F | CGGGCAGATTCTAGACCTCCTG | Fields, Popovic *et al.* 1992 |
|  |  | PCTA-614R | CGATGATCTTGGAGCATTCCCAC |  |
|  | O1 | pTcpA-647R | TTACCAAATGCAACGCCGAATG | Huq *et al.* 2006 |
|  |  | pTcpA-72F | CACGATAAGAAAACCGGTCAAGAG |  |
|  | O139 | pTcpA-477R | CGAAAGCACCTTCTTTCACGTTG | Huq *et al.* 2006 |
|  |  | pTcpA-72F | CACGATAAGAAAACCGGTCAAGAG |  |
| *V. mimicus* | ITS | PVM-F1 | TTTAAGTGCATTCGRTGAGTGC | Huq *et al.* 2006 |
|  |  | PVCM-R1 | AGTCACTTAACCATACAACCCG |  |
| *V. alginoyiticus* | Collagenase-targeted | VA-F | CGAGTACAGTCACTTGAAAGC | Di Pinto *et al.* 2004 |
|  |  | VA-R | CACAACAGAACTCGCGTTACC |  |
| *V. vulnificus* | ITS | VV1 | GACTATCGCATCAACAACCG | Marion Fischer-Le Saux *et al.* 2012 |
|  |  | VV2R | AGGTAGCGAGTATTACTGCC |  |
