# Supplementary material for: Occurrence and Diversity of Clinically Important Vibrio Species in the Aquatic Environment of Georgia
Source: Front Public Health. 2015 Oct 13;3:232. doi: 10.3389/fpubh.2015.00232 (PMC4603242; doi:10.3389/fpubh.2015.00232)
Supplement: Supplementary file 4 [file Image_1.PDF]

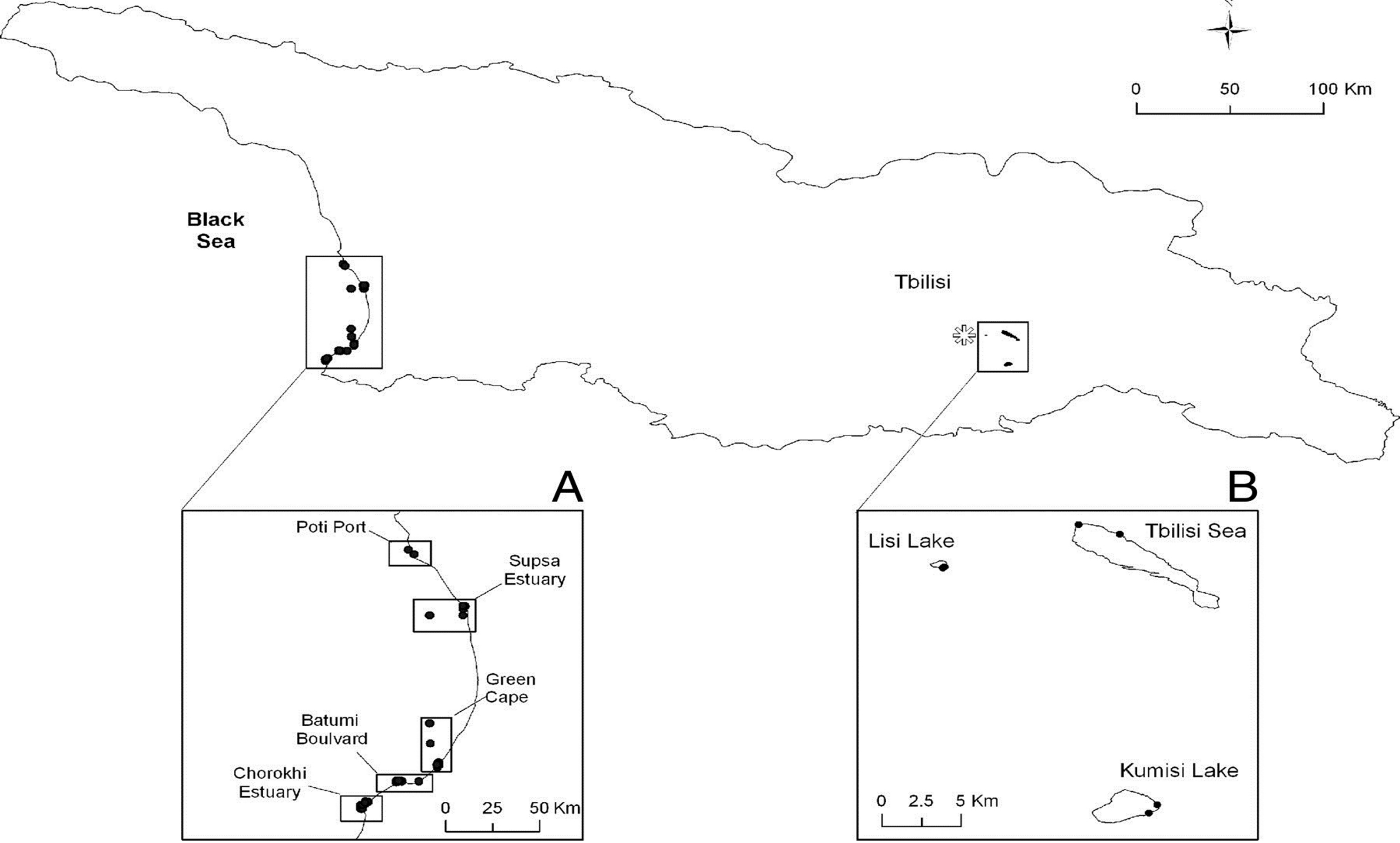

Supplementary Figure 1 | Map of Georgia including sampling sites. A map of Georgia showing the sites of water sample collection. Inset (**A**) shows collection sites along the Black Sea coast. Inset (**B**) shows collection sites among inland water sources.
